# Wounded healer nurses: a qualitative content analysis of the positive traits of nurses affected by chronic cardiovascular disease

**DOI:** 10.1186/s12912-024-02124-3

**Published:** 2024-07-09

**Authors:** Mahdi Nabi Foodani, Masoumeh Zakerimoghadam, Shahrzad Ghiyasvandian, Zahra Abbasi Dolatabadi

**Affiliations:** Department of Medical-Surgical Nursing, School of Nursing and Midwifery, Tehran University of Medical Sciences, Tehran, Iran

**Keywords:** Chronic illness, Nurse, Positive traits, Qualitative content analysis, Wounded healer

## Abstract

**Background:**

The wounded healer concept refers to healthcare providers who, in the past, have had similar experiences to those of their clients and now draw on these challenging experiences to assist their clients. This study explored the positive traits of nurses with chronic cardiovascular diseases who transitioned to wounded healers.

**Methods:**

A qualitative content analysis study was conducted within hospitals in Tehran, Iran, between November 2023 and March 2024. Sampling was conducted using a purposive sampling method in accordance with the study objectives and inclusion criteria. The data were collected through semi-structured face-to-face interviews. Twenty-three participants, comprising 16 females and 7 males, participated in the interviews. Data analysis was conducted by employing a qualitative content analysis approach, including creating codes, subcategories, generic categories, and main categories. MAXQDA v20 software was utilized to facilitate the analysis process.

**Results:**

The data analysis revealed one main category that aligned with the research question: the positive traits of a wounded healer nurse, consisting of three generic categories: (1) traits related to interpersonal and professional relationships; (2) traits related to the professional dimension; and (3) traits related to the personal dimension. wounded healer nurses demonstrate positive traits that enhance patient care.

**Conclusions:**

The findings of this study have important implications for nursing practice and education. By identifying the positive traits exhibited by nurses as wounded healers affected by chronic cardiovascular diseases, nursing programs can emphasize and strengthen these qualities to convert challenges into opportunities and bridge the theory-practice gap.

**Supplementary Information:**

The online version contains supplementary material available at 10.1186/s12912-024-02124-3.

## Background

The concept of the wounded healer, which originated from ancient Greek myths, was first articulated by Carl Jung to elucidate a phenomenon within the psychologist-client relationship [1]. Jung posited that wounded healers leverage their own painful experiences to cultivate insight and adaptability, thereby creating an environment conducive to effective therapeutic interventions [2]. This concept refers to healthcare providers who, in the past, have had similar experiences to those of their clients and now draw on these challenging experiences to assist their clients. Conti-O’Hare [3] introduced the concept of the “wounded healer” into the nursing discipline and developed the theory of the “nurse as a wounded healer”. She emphasized that in the face of trauma (physical, emotional, psychological, and spiritual), nurses can adopt either effective or ineffective coping strategies. Individuals employing ineffective coping strategies may act as “walking wounded” and project their own struggles onto patients and colleagues, demonstrating less empathy. In contrast, nurses who select effective coping strategies can grow from their pain, transform their experiences into healing, and become wounded healers [4, 5].

Despite its significance, limited literature has delved into the phenomenon of the wounded healer within nursing. For instance, a study examined nurses’ experiences with COVID-19, encompassing their recovery, return to work, and transformation into wounded healers. These nurses, after battling COVID-19, gained profound insights into patient conditions, consequently enhancing the quality of nursing care [4]. Similarly, Ladds et al. [6] explored the enduring impacts of COVID-19 on healthcare providers, revealing opportunities for personal growth and heightened caregiving efficacy. In an oncology nursing study, the concept of the “wounded healer” was introduced, emphasizing the mutual suffering experienced by both nurses and patients. This research proposed that acknowledging the physical and emotional distress of cancer patients could form the foundation of comprehensive care, foster compassion and empathy, and thereby enhance the standard of nursing care [7]. Similarly, a qualitative study conducted in Iran explored the experiences of nurses caring for patients with COVID-19. These nurses encountered numerous personal and professional challenges, including excessive workload, fear, anxiety, worry, and compassion fatigue. Despite these challenges, nurses persist in providing care, embodying the essence of the wounded healer concept [8]. An editorial commentary further delved into the concept of the wounded healer in nursing and its implications. It has been argued that personal experiences, particularly those involving suffering, have the potential to enrich the nurse‒patient relationship. The commentary proposed that acknowledging and reflecting on one’s own wounds can transform them from burdens into resources for healing [9].

Existing evidence highlights the pivotal role of nurses’ traits in shaping the quality of patient care. The transformation into a wounded healer can significantly influence the personal, social, and professional dimensions of nursing practice. Given the potential impact on patient outcomes and satisfaction, wounded healers represent invaluable assets to healthcare organizations [10–13].

In sociology and anthropology, it has been suggested that personal characteristics can significantly influence human interaction and behavior [14]. By recognizing the positive attributes of wounded healers, it is possible to enhance the dynamics of nurse‒patient interactions. However, such investigations are rarely conducted in nursing, leading to a knowledge gap regarding the positive traits that wounded healers may possess.

Understanding the positive traits of wounded healer nurses is essential for enhancing care programs, emphasizing the positive attributes that may arise from illness experiences. Strengthening these qualities can convert challenges into opportunities, aligning with the nursing theory of the “nurse as a wounded healer” and bridging the theory-practice gap. Despite the growing recognition of the wounded healer concept in nursing, there remains a critical gap in research specifically exploring the positive traits of nurses as wounded healers. This gap hampers our understanding of how nurses with chronic cardiovascular illnesses leverage their own experiences to enhance patient care, particularly in the context of providing care to patients with similar ailments. Identifying and understanding these positive traits is essential for developing targeted interventions and support systems to empower nurses in their roles and improve patient outcomes. Therefore, this study aimed to address this research gap by identifying and understanding the positive traits exhibited by nurses with chronic cardiovascular illnesses who provide care to patients with similar ailments.

## Methods

### Study design

This research utilized a qualitative content analysis design. These types of studies aim to discover and understand phenomena, processes, or the perspectives, points of view, and worldviews of the people involved [15]. This study aimed to address the research gap in exploring the positive traits of nurses as wounded healers by identifying and understanding the positive traits exhibited by nurses with chronic cardiovascular illnesses who provide care to patients with similar conditions. The study adhered to the Standards for Reporting Qualitative Research (SRQR) reporting guidelines [16] (Additional file 1).

### Participants and sampling

This study was conducted within hospitals in Tehran, Iran, between November 2023 and March 2024. Twenty-three participants, comprising 16 females and 7 males, with a mean age of 45.6 ± 10.9 years (range 26–62) and work experience of 20.1 ± 8.4 years (range 4–32), participated in the interviews (Table 1).

**Table 1:** Demographic information of participants in

participant	Sex	Work experience (year)	Degree	Chorionic cardiovascular condition
1	Female	23	B.Sc.	Cardiomyopathy
2	Female	26	Ph.D.	Dysrhythmia
3	Female	27	B.Sc.	Hypertension and unstable angina
4	Male	30	Ph.D.	Myocardial infarction
5	Male	20	Ph.D.	Diastolic hypertension
6	Female	23	B.Sc.	Having a history of coronary artery bypass graft surgery
7	Male	8	B.Sc.	Mitral valve replacement
8	Female	27	M.Sc.	Tachyarrhythmia
9	Female	20	M.Sc.	Having internal cardiac defibrillator
10	Male	32	Ph.D.	Hypertension and Arrythmia
11	Female	4	B.Sc.	Varicose veins
12	Male	6	B.Sc.	Mitral regurgitation
13	Female	18	Ph.D.	coronary heart disease
14	Female	25	B.Sc.	Rheumatic heart disease
15	Male	26	B.Sc.	Heart failure
16	Male	7	B.Sc.	Congenital heart disease
17	Female	27	B.Sc.	Heart attack
18	Female	24	B.Sc.	Dilated cardiomyopathy
19	Female	11	B.Sc.	Hypertension
20	Female	23	B.Sc.	Cardiomegaly
21	Female	20	B.Sc.	Pulmonary heart disease
22	Female	9	B.Sc.	Dysrhythmia
23	Female	28	B.Sc.	Aortic aneurysm

Sampling was conducted using a purposive sampling method in accordance with the study objectives and inclusion criteria [17]. The inclusion criteria for participants to be included in the study were as follows: (i) aged > 22 years; (ii) held a nurse certificate; (iii) had chronic cardiovascular disease; and (iv) accepted and adapted to the disease (we used self-reported measures of acceptance and adherence from participants, along with objective indicators of disease control). The exclusion criterion for the study was the participant’s unwillingness to complete the interview. Participant selection was conducted by trained research team members (MNF and ZAD), who distributed invitations to nurses either face-to-face or via telephone. The researchers explained the study’s purpose, precautions, and confidentiality principles to potential participants through telephone or face-to-face conversations. Participation in the study was voluntary, and all participants provided written informed consent before participation. Nurses with chronic cardiovascular diseases were selected as the specific population due to their unique perspective and first-hand experience living with chronic illness while providing care to patients with similar conditions. Additionally, the research focused on nurses afflicted with chronic cardiovascular conditions due to the elevated prevalence of cardiovascular diseases among nurses and their accessibility. This selection provides valuable insights into the positive traits that may emerge from such shared experiences. Sampling continued until data saturation was achieved. Data saturation was achieved when no new information or categories emerged from the interviews, indicating that a comprehensive exploration of the positive traits had been achieved. The saturated sample size of 23 participants in this study aligns with similar studies and descriptions [18]. After completing the interviews with the initial 20 participants, additional interviewees were recruited to ensure genuine data saturation, aiming for the highest level of comprehensiveness and quality in the study. Notably, 27 individuals were invited to participate in this study, but 4 of them declined due to personal reasons.

### Determining the interview outline

To develop the interview guide as recommended by Kallio et al. [19], a five-step process was followed: (i) identifying the prerequisites for using semi-structured interviews, which involved determining the suitability and relevance of this method for the research context; (ii) gathering and utilizing existing knowledge; (iii) drafting the preliminary semi-structured interview guide; (iv) conducting a pilot test of the guide; and (v) finalizing and presenting the complete semi-structured interview guide. Initially, an interview framework was created based on the research objectives and a thorough review of the relevant literature. This was followed by pre-interviews with two nurses, which, after a group discussion, led to the establishment of the final interview framework. Two researchers (MNF and ZAD) then separately conducted the trial runs with two additional nurses who were not part of the main study. These pre-interviews informed the refinement and finalization of the formal interview framework.

### Data collection

Data collection was conducted through semi-structured face-to-face interviews using a semi-structured topic guide (Table 2). This method allows researchers to delve deeply into conversations and responses, exploring them in depth and multidimensionally [20, 21]. The location and timing of the interviews were coordinated with the participants based on their preferences. Most of the interviews were conducted in the staff room after their shifts, and some were conducted in the hospital’s coffee shop. Initially, interviews began with general questions such as “Can you describe a typical day in your role as a nurse?” Subsequently, based on the participants’ responses, more detailed questions were asked, such as “What qualities do you believe distinguish you from other nurses after being diagnosed with your illness?” Exploratory questions such as “what” and “how” were used to gather further information. The duration of the interviews ranged from 30 to 60 min, depending on the interview circumstances. When needed, the interviewer utilized questioning, rhetorical questioning, and repetition methods to validate the participants’ answers and ensure comprehension. During the interviews, the main researcher (MNF) conducted each interview while actively observing, listening, and recording the interviewees’ expressions, voices, and intonation. Additionally, any uncertain information was clarified and verified to enhance the accuracy of the data. The interview guide comprised open-ended questions, enabling participants to fully articulate their viewpoints, perceptions, and experiences. At the onset of each interview, the participants were requested to provide their information, educational degree, and work experience. Throughout the interviews, the researcher (MNF) maintained a linguistically and nonjudgmentally neutral stance to facilitate observation and recording. All interviews were recorded using an audio recording device and transcribed by the MNF after completion of the interview.

**Table 2:** Interview guide for semi-structured interviews

Type of question	Questions
Greeting and Introduction	Introduce the interviewer and explain the study’s purpose.
	Ensure confidentiality and obtain consent for recording.
	How old are you, and how many years have you been working as a nurse? how many years of experience do you have working as a nurse? What is your cardiovascular disease? How long are you affected by this disease?
Background Information	Can you tell me about your professional background and your current role as a nurse?
	Can you describe a typical day in your role as a nurse?
Personal Experience with Chronic Cardiovascular Disease	Can you describe your experience with chronic cardiovascular disease?
	How has your condition impacted your daily life?
Impact on Professional Role	How has living with a chronic cardiovascular disease influenced your approach to patient care?
	Can you provide specific examples where your personal experience with illness has positively affected your interactions with patients?
Development of Positive Traits	What qualities do you believe distinguish you from other nurses after being diagnosed with your illness
	How have these traits benefited your professional practice
Reflection and Advice	Looking back, what advice would you give to other nurses who are providing care to patients with chronic cardiovascular disease?
Closing Remarks	Is there anything else you would like to share about your experience as a wounded healer nurse?
	Thank you very much for your time and insights. Your contributions are invaluable to this research

### Data analysis

This study used conventional inductive content analysis to explore and understand the positive traits exhibited by nurses afflicted with chronic cardiovascular illnesses while simultaneously accepting and adapting to their conditions and presently delivering care to patients with similar ailments. This approach involves systematically coding and identifying patterns in textual content without consideration of the methods of data collection. This type of data interpretation aids in achieving a deep understanding of human experiences and perceptions [22]. The unit of analysis consisted of recorded interview transcripts. MNF, ZAD, and MZ meticulously read through the interview text multiple times to immerse themselves in the data. To conduct content analysis, the steps outlined by Elo and Kyngäs were followed, including creating codes, subcategories, generic categories, and main categories [23]. All these steps were individually performed by three researchers, MNF, ZAD, and MZ, and after completing the individual analyses, a consensus meeting was held to organize the data. MAXQDA v20 software was utilized to facilitate the analysis process.

In the initial phase, researchers MNF, MZ, and ZAD meticulously reviewed the recorded data multiple times, thoroughly examined the transcribed text, and identified any transcription errors for prompt rectification. This process involved extracting pertinent content from the transcript and annotating the interviewer’s expressions in brackets based on the participants’ verbatim statements. Subsequently, meaningful units were distilled from the transcribed text by condensing and summarizing recurring words or sentences as appropriate, employing inductive analysis by MNF, ZAD, and MZ. Following the completion of open coding, MNF, ZAD, and MZ compiled a list of subcategories and generic categories. This was followed by a comprehensive review of all codes, subcategories, and generic categories by the researchers, as emerged from the transcripts. Given the differing perspectives among team members, the iterative process continued until a consensus was reached. The findings of the content analysis process are reported in Table 3 (Additional file 2).

### Trustworthiness

In line with Guba and Lincoln’s [24] criteria for trustworthiness in qualitative research, this study employed various strategies to establish credibility, dependability, confirmability, and transferability. The researchers’ preunderstanding influenced the data collection and analysis, guiding the development of the research questions and data interpretation. To mitigate bias and ensure rigor, they maintained a reflexive journal, used triangulation by involving multiple researchers, and conducted member checking with participants to confirm findings.

Credibility was ensured through member checking and triangulation of the data sources. Comprehensive descriptions were provided to mitigate the risk of overlooking vital information, thus enhancing the meaningful interpretation of the data. Additionally, a member-check process was conducted, wherein participants received interview transcripts and extracted codes for comparison with the researchers’ interpretations, providing valuable feedback. Triangulation, involving independent analysis by three researchers (MNF, ZAD, and MZ), was another method used to ensure trustworthiness. Furthermore, peer checking was employed to enhance credibility, with codes and categories evaluated by ShGh, an experienced qualitative researcher [25].

To establish dependability, an audit trail was meticulously maintained, documenting all stages of the research process, from data collection to analysis and interpretation. The study methodology was comprehensively described, aiming to facilitate replication by other researchers. By maintaining detailed records and providing clear methodological descriptions, the study sought to enhance the dependability of its findings.

Confirmability was achieved by maintaining reflexivity and acknowledging the researchers’ potential biases throughout the study. This ongoing reflection ensured transparency and minimized the influence of subjective biases on the research process and findings. Additionally, transferability was addressed through the provision of rich, detailed descriptions of the research context, methods, and findings. By presenting a comprehensive understanding of the phenomenon under investigation, readers could assess the applicability of the findings to their own contexts. This approach facilitated comparison and extrapolation of the research findings to similar situations, thereby enhancing the study’s transferability and overall credibility [26]. By adhering to these principles, we aimed to enhance the trustworthiness and credibility of our research findings.

### Ethical considerations

This study was conducted in accordance with the Helsinki Declaration [27]. The Research Ethics Committee of the Faculty of Nursing and Midwifery at Tehran University of Medical Sciences approved this study (ethical code: IR.TUMS.FNM.REC.1402.064). The researchers explained the study’s purpose, precautions, and confidentiality principles to the participants. All participants were informed about the study details, and informed consent was obtained from them. Participants had the option to withdraw from the study at any time they desired. Participant anonymity was ensured by removing any identifying information, such as names or specific locations, during the transcription and analysis phases. Additionally, during the preparation of this manuscript, all identifying details that could compromise anonymity were either removed or generalized to ensure confidentiality. To preserve participant privacy, interview audio files are encrypted and stored with the principal researcher, and after a specified period, the audio records are deleted. Participants were informed that their decision to participate in this research would have no effect on them. Additionally, protective measures to maintain their privacy were described to them before the beginning of the interview. Participants did not receive any financial compensation or other benefits for participating in the study. The decision to participate was voluntary, and participants were informed that their involvement would solely contribute to advancing research in the field.

## Results

Twenty-three participants, comprising 16 females and 7 males, participated in the interviews. A total of 632 codes were extracted from the analysis of 23 interview transcripts. The data analysis revealed that the main category that emerged that aligned with the research question was the positive traits of a wounded healer nurse, which consisted of three generic categories: traits related to interpersonal and professional relationships, traits related to the professional dimension, and traits related to the personal dimension (Additional file 2).

### Traits related to interpersonal and professional relationships

This generic category includes two subcategories: appropriate communication with patients and their families and constructive professional relationships with colleagues. The participants in the current study highlighted that wounded healer nurses possess the unique ability to cultivate strong interpersonal and professional relationships, driven by their personal experiences with illness. This background enhances their empathy and understanding, allowing them to deeply connect with patients and their families and providing comfort and reassurance through active listening and compassionate communication. These nurses’ skills extend to their professional interactions, where they are collaborative, supportive, and attuned to the needs of their colleagues, often taking on mentorship and leadership roles. Their empathy and communication skills make them effective at resolving conflicts, fostering a positive work environment, and ultimately contributing to improved patient care and team dynamics.

### Appropriate communication with patients and their families

Participants in the study reported that after their diagnosis, they saw a marked improvement in their communication with patients and families, recognizing it as essential for quality healthcare. For instance, one nurse stated:

…When I disclose to the patient who I also have a heart condition, they tend to trust me more and establish a connection with me more readily… (Participant 4).

Participants stressed the importance of clear, concise, and accurate information while being attuned to the emotional and psychological needs of patients and their relatives. Nurses exhibiting traits of wounded healers, such as active listening, empathy, and responsiveness, faced fewer conflicts. Using jargon-free language ensured that patients and families fully understood diagnoses, treatment options, and care plans. This holistic approach not only improved daily interactions but also facilitated better therapeutic communication, reduced misunderstandings, and improved healthcare outcomes.

### Constructive professional relationships with colleagues

Participants in the current study reported that their diagnosis and adaptation to the disease led to more constructive relationships with their healthcare colleagues. In one of the interviews, a nurse stated:

…I extensively studied my own illness, which enabled me to better understand the medical literature and language regarding this disease. Consequently, it improved my communication with medical doctors… (Participant 18).

These nurses observed significant improvements in collaboration, communication, and mutual support, which facilitated smoother workflows, encouraged knowledge sharing, and created a more cohesive and efficient healthcare environment. These healthcare professionals, influenced by their personal experiences, actively promoted a culture of teamwork and shared goals, ultimately enhancing patient care and overall job satisfaction.

### Traits related to the professional dimension

This generic category comprised five subcategories, including having a strong professional identity, providing transcendent care, paying more attention to patient education and empowerment, having a deeper understanding of patients, and serving as mentors and role models for colleagues and nursing students. The current study identified traits related to the professional dimension of wounded healer nurses, highlighting their profound commitment to nursing. These traits include a strong professional identity, transcendent care, an emphasis on patient education and empowerment, a deep understanding of patients’ needs and role modeling of mentorship and leadership. Wounded healer nurses exemplified resilience, empathy, and integrity, making significant contributions to patient care, colleague support, and the advancement of nursing education and practice.

### Having a strong professional identity

The participants in the present study revealed that their disease diagnosis did not weaken their professional identity but rather enhanced and refined it. In this regard, one of the nurses admitted:

… Since I myself became a recipient of nursing services, I am more in love with nursing than ever before… (Participant 6).

Facing personal health challenges led them to discover profound resilience, which strengthened their dedication to nursing. Adversity fueled their passion for the profession, igniting a deeper sense of purpose and commitment. They transformed their experience of illness into a source of strength and inspiration, gaining a heightened appreciation for the privilege of being a nurse. This perspective enriched their fulfillment and reaffirmed their identity within the nursing community.

### Providing transcendent care

Participants emphasized that providing transcendent care involves moving beyond routine practices to establish profound connections and understanding with patients. In this regard, one of the participants stated:

… My negative experience of not being listened to during my hospitalization has led me to pay more attention to the preferences and desires of my patients… Patient-centered care is beyond routine care (Participant 22).

Wounded healer nurses, drawing from their personal experiences with illness, create care plans that prioritize holistic well-being. They deeply respect patients’ beliefs and preferences, ensuring that every interaction maintains the individual’s dignity. Through empathetic engagement and personalized interventions, these nurses foster an environment where patients feel seen, heard, and valued, building trust and empowerment that surpasses conventional healthcare boundaries.

### Paying more attention to patient education and empowerment

Participants stated that their professional knowledge of controlling and managing their illness was very helpful. Therefore, they are highly focused on educating and empowering their patients while providing care. For example, one nurse said:

…I was a nurse and knew a lot about the disease, but the patient is not like that… Empowering patients in any way possible can be a factor in preventing many disease complications… (Participant 1).

The participants emphasized the importance of educating and empowering patients. They leverage their professional knowledge to provide comprehensive education, help patients understand their conditions and actively participate in their care. This approach not only enhances patient outcomes but also fosters a sense of empowerment and self-efficacy among patients.

### Deeper understanding of patients

Participants’ first-hand experience of illness gave them profound insight into patients’ lived experiences, fostering a deeper understanding of their conditions and perspectives. One of the participants expressed:

Now I understand what it means when my patient says they cannot climb two more steps… (Participant 9).

This heightened awareness allowed them to empathize more fully and comprehend patients’ concerns with greater clarity. Through this empathetic connection, wounded healer nurses are better equipped to tailor their care to meet individual needs and preferences, enhancing the quality and effectiveness of patient-centered care.

### Serving as mentors and role models for colleagues and nursing students

Fulfilling the role of mentor and serving as role models for novice nurses and nursing students was another characteristic of wounded healer nurses mentioned by the participants. For example, one nurse said:

Even though I sometimes have dyspnea, I do not let the work in my department stay on the ground… After seeing these conditions, one of the young nurses told me that you are a professional role model for me… (Participant 8).

Some of the participating nurses believed that by disclosing their own illness status to novice nurses and nursing students, as well as demonstrating their enthusiasm and passion for nursing, they inspire and motivate these young colleagues. This mentoring role helps in nurturing the next generation of nurses and fostering a supportive and learning-oriented environment within healthcare settings.

### Traits related to personal dimension

This category encompasses three subcategories: increased resilience and adaptation, greater empathy and compassion, and posttraumatic growth, highlighting the transformative power of healing from within. The final generic category identified in the study pertains to the personal traits of wounded healer nurses, revealing the qualities that define their inner landscape. These nurses exhibit resilience that goes beyond endurance, allowing them to thrive amid challenges. Their empathy and compassion, deepened by their own suffering, enable them to form genuine connections with patients. Their journey through adversity fosters personal growth, offering new perspectives, insights, and wisdom that enrich their practice.

### Increased resilience and adaptation

The interviews with participants highlighted a recurring theme of resilience, flexibility, and enhanced adaptability among wounded healer nurses. Their experiences with illness significantly influenced their approach to workplace challenges, increasing their ability to recover from setbacks, navigate obstacles, and embrace change with confidence and resourcefulness. This enhanced resilience and adaptability, described as transformative, empowered them to tackle professional challenges more effectively. Participants illustrated how adversity strengthened their resolve and equipped them with the tools and mindset needed to thrive in the demanding healthcare environment.

### Increased empathy and compassion

Most nurses reported feeling more empathy and compassion after experiencing their own illness. This subcategory highlights a profound shift in wounded healer nurses’ emotional landscape, forged through adversity and healing. In one interview, a nurse admitted:

… Empathy and sympathy do not occur until a person experiences pain themselves… (Participant 2).

These nurses develop heightened sensitivity to others’ pain and struggles, cultivating deep empathy that allows them to truly connect with their patients. Their personal encounters with suffering and resilience provide unique insights into the human condition, fostering authentic compassion. This empathy shapes their practice, guiding their interactions with warmth, understanding, and kindness and transforming empathy into a powerful catalyst for healing and connection.

#### Post traumatic growth

In interviews with wounded healer nurses, the subcategory of posttraumatic growth emerged as a narrative of transformation. Nurses candidly share their journeys through adversity, revealing how trauma became moments of personal evolution. Through introspection and perseverance, they navigated suffering, emerging with newfound insights and strengths. Posttraumatic growth is a journey of self-discovery and empowerment that leads to a deeper understanding of oneself and others, fostering purpose and resilience beyond past trauma. Their stories inspire hope, illustrating the human capacity to find meaning and growth in adversity.

## Discussion

Based on the study results, contracting and adapting to an illness can create new traits for nurses. These characteristics are classified into three generic categories: traits related to interpersonal and professional relationships, traits related to the professional dimension and traits related to the personal dimension. Although this study was conducted in Iran, some of the findings are similar to those in other countries. A parallel investigation by Piredda et al. [4] demonstrated that nurses, post-COVID-19 contractions, evolve into what can be termed “wounded healers”, thereby enhancing their therapeutic rapport with both patients and peers. Similarly, psychological inquiries have elucidated how wounded healers transcend their personal afflictions, leveraging these experiences to foster deeper therapeutic alliances with their clientele [28]. In alignment with these broader insights, our study corroborates that nurses, following illness contraction and adaptation, exhibit heightened relational adeptness with both patients and colleagues. Consequently, our study augments the existing corpus of scientific knowledge in this domain.

Moreover, the findings also reveal that possessing a robust professional identity is another hallmark of wounded healer nurses. Similar investigations have shown that some nurses, following traumatic physical and psychological events, embark on a reevaluation of the esteemed tenets inherent in the nursing profession. For instance, in a study conducted by Johnstone et al. [29], nurses who encountered traumatic events in disasters and crises acknowledged experiencing a sense of pride in their nursing profession and rediscovered the core professional values despite the significant pressures they encountered. In another inquiry, nurses afflicted by COVID-19 expressed that, subsequent to the initial crisis precipitated by the pandemic, they attained a renewed recognition of the foundational values of nursing and took pride in their nursing identity [4].

The results of the current study indicate that one of the traits of wounded healer nurses is the provision of transcendent care. These findings align with those of the study by Cuseglio [30], which suggested that all traumas and traumatic events during childhood can significantly influence the formation of a therapist’s professional personality and transform the care he or she provides. According to the findings of this study, the care that wounded healer nurses can provide is of greater quality than routine care. In another study conducted on Japanese physicians, illness and recovery led to changes in their thinking and behavior regarding the provision of medical services to their patients. The results of this study suggest that some of these physicians seek to acquire new medical knowledge and provide superior services to their patients, which is consistent with the findings of the current study. However, some of the results of this study showed that some physicians exhibited signs of reduced self-confidence in their profession, which contradicts the findings of the present study [31]. Nevertheless, considering that this study was conducted on physicians whose role involves accurate diagnosis and prognosis for patients, some degree of justification for this contradiction can be made.

Based on the results, another prominent feature repeatedly emphasized by wounded healer nurses is their heightened focus on educating and empowering their patients. Two studies conducted on former inmates revealed that postliberation individuals, who function as wounded healers, devote themselves to educating and empowering current prisoners and those newly released to reintegrate them into normal life [32, 33]. While the context of these studies differs from the nursing context of the present study, they were conducted within the framework of the wounded healer concept, and their findings resonate with the present study.

Additionally, a notable trait of wounded healers is their deeper understanding of patients’ conditions. Similarly, in the study by Zerubavel and Wright [34], this attribute was highlighted among wounded healers. Although this study also emphasized the necessity of supporting wounded healers as vulnerable individuals and understanding their conditions, it falls outside the scope of the present study. In numerous other studies, the development of a deeper understanding following traumatic experiences has been noted for experiencers [31, 32, 35].

Furthermore, traces of the wounded healer concept can even be found in Persian poetry and the literature. For instance, in one of his poems, Omid SabaghNo [36, p123], a Persian-language poet, wrote, “I have suffered the pain of love that only the afflicted understand, the man understands the meaning of grief when he loses a bet”. Although this poetry may not directly relate to the concept of wounded healers, it implies that individuals can only fully comprehend an event after personal experience with it.

Some participants identified themselves as mentors and role models for their nursing peers and students. In a study conducted by Powell et al. [37], the experiences of nurses subjected to peer violence were examined. The findings indicated that these nurses transitioned into wounded healers within their profession, assuming the roles of mentorship and support for novice colleagues. Although the trauma encountered in this study pertained to psychological distress, which is distinct from the findings of the present investigation, it also underscores the significance of mentorship and role modeling for wounded healers, aligning with the focus of the present study. Furthermore, within social work research, leveraging the experiences of wounded healers for student training and education has been highlighted. For instance, Murphy [38] identified childhood trauma as a motivating factor for individuals pursuing careers in social work. These individuals may subsequently serve as active mentors, imparting their experiences to students. The findings of this study resonate with those of the present study.

Increased resilience and adaptation to challenges are also characteristic features of wounded healers. However, it has been noted in most studies that resilience increases in individuals involved in a challenging event following a successful coping experience [4, 7, 39]. However, in a study conducted by Wheeler [40], wounded healers were identified as a dilemma for supervisors. On the one hand, the unique characteristics of these individuals have been highlighted, while on the other hand, their increased vulnerability to workplace pressures has posed challenges for supervisors. The second part of this study, which indicates the heightened vulnerability of injured healers to workplace challenges, differs from the findings of the present study. However, since this study was conducted on upper-level managers rather than on the wounded healers themselves, it is justified because the perspective of managers may differ from that of wounded healer nurses.

One of the most prominent traits of wounded healers, according to participants in the present research, is their heightened compassion and empathy toward their patients compared to other colleagues. In the study by Vincent and Corso [7], after experiencing various existential events throughout their professional careers, oncology nurses transformed into wounded healers, developing a compassion identity. These nurses exhibit a high level of empathy and compassion. According to the results of a study where a physician became a patient, after experiencing illness, the physician understood patients’ experiences better, demonstrated greater empathy toward all patients, and showed increased compassion and kindness in their interactions with them [41]. The findings of these studies align with and resonate with the present research.

Based on the findings of this study, increased compassion and empathy stand out as pivotal attributes among wounded healer nurses following their diagnosis of the disease and encounters with challenges. These outcomes resonate with Travelbee’s [42] concept of “therapeutic use of self,” which emphasizes the deliberate deployment of one’s personality and understanding of human dynamics to enhance nursing interventions. Travelbees underscore the importance of self-insight, a deep understanding of human behavior, and the ability to interpret behaviors within clinical contexts, influenced by personal beliefs about illness, suffering, and death. This framework highlights how nurses can integrate personal insights into their professional practice, foster meaningful connections with patients and enhance therapeutic outcomes.

According to the literature, a common trait among all wounded healers, regardless of the mechanism of injury they have faced, is posttraumatic growth and resilience. For example, McCarthy et al. [43] indicated in their study that after traumatic incidents, both physical and psychological posttraumatic growth can create greater capacities for individual development. In the present study, wounded healer nurses also experienced posttraumatic growth. Another study reviewed the experiences of an individual who had recovered from addiction and helped others [44]. According to the results of this study, posttraumatic growth was one of the most significant traits that individuals acquired in various domains after overcoming addiction. Although this study was conducted on a different population than the present study, its results suggest that individuals can experience growth after surviving a traumatic event. These findings are consistent with the present study.

This study was conducted within the context of Iran, which may differ to some extent from other countries. Future research should include wounded healer nurses affected by various physical and psychological illnesses in a multicounty context. Additionally, future quantitative studies could be designed based on the findings of the present study to investigate the characteristics of wounded healer nurses, thus enhancing the generalizability of the results.

### Implication for nursing

The positive attributes of wounded healer nurses identified in the present qualitative study have significant implications for the nursing profession. In nursing education, these findings suggest the potential for wounded healer nurses to serve as mentors and role models, transmitting their positive qualities to new nurses and nursing students. Within clinical practice, leveraging these attributes could lead to enhanced care programs tailored to the unique perspectives of wounded healer nurses, thereby improving patient outcomes. Furthermore, in nursing management, understanding and utilizing these traits may inform the development of more supportive policies and practices, ensuring adequate support for this vulnerable population of nurses. Finally, in nursing research, the identified positive attributes provide a rich foundation for further exploration, potentially leading to the development of innovative interventions and strategies to enhance nursing care delivery and improve patient experiences.

## Conclusion

The findings of this study contribute to an enhanced understanding of the attributes of wounded healer nurses. These unique and quality-enhancing traits of wounded healer nurses can be prioritized in nursing student education programs and continuous education initiatives for nursing staff, serving as educational content to empower nurses. Nursing administrators and decision-makers can leverage wounded healer nurses’ assistance, utilizing role modeling techniques, to reinforce these attributes among other nursing professionals. By harnessing the abilities of wounded healer nurses, who exhibit exceptional attributes according to this study, nursing care at the bedside can be improved, leading to increased patient satisfaction—the primary beneficiaries of nursing care. Wounded healer nurses represent valuable human capital in the nursing and healthcare systems, offering distinct experiences. When devising nursing action plans, nursing managers can optimize the utilization of these capacities and specific attributes. Furthermore, awareness of these attributes can inform the integration of theory into clinical practice, enhancing nursing care plans through the incorporation of the perspectives and attributes of wounded healer nurses. The attributes of wounded healer nurses in nursing lay the groundwork for designing future research aimed at enhancing nursing programs with a theoretical framework centered on nurses as wounded healers. Researchers can give heightened consideration to these attributes in their investigations.

### Limitations and strengths

Although this study offers comprehensive insight into the positive traits demonstrated by wounded healer nurses, it also has some limitations. First, during face-to-face interviews, the interviewer endeavored to recall and document participants’ facial expressions. However, capturing and recording all expressions and movements proved challenging, potentially leading to oversight. Second, the interpretation and construction of the interviews may have influenced the data collection and analysis, potentially resulting in a less thorough exploration of certain content. Additionally, the translation of quotes from Persian to English introduces the possibility of nuanced differences in meaning between the original and translated versions. Despite these limitations, this study offers unique insights into the positive traits demonstrated by wounded healer nurses experiencing chronic cardiovascular disease, highlighting how personal adversity can profoundly enhance empathetic connections and therapeutic outcomes in nursing practice.

### Electronic supplementary material

Below is the link to the electronic supplementary material.

Supplementary Material 1

Supplementary Material 2

## Data Availability

All the raw data (including participants’ voice files and the texts of the interviews) will be confidential and will not be able to share publicly. However, the codes that emerged during the current study are available from the corresponding author upon reasonable request.

## Abbreviations

CABG: coronary artery bypass graft
SRQR: Standards for Reporting Qualitative Research
