# Supplementary material for: Wounded healer nurses: a qualitative content analysis of the positive traits of nurses affected by chronic cardiovascular disease
Source: BMC Nurs. 2024 Jul 9;23:465. doi: 10.1186/s12912-024-02124-3 (PMC11232157; doi:10.1186/s12912-024-02124-3)
Supplement: Supplementary file 2 — Supplementary Material 2 [file 12912_2024_2124_MOESM2_ESM.docx]

**Table 3- The summary of the process of data abstraction from the nurse's interviews**

| Codes | Sub-categories | Generic Categories | Main categories |
| --- | --- | --- | --- |
| -Rapport and trust with patients  -Better communication with patients' families  -Engage families in care | Appropriate communication with patients and their families | Traits related to Interpersonal and professional relationships | **Positive traits of Wounded Healer Nurses** |
| -Collaborate with team effectively  - Foster supportive colleague relationships  -Communicate openly and respectfully | Constructive professional relationships with colleagues |  |  |
| -Dedication to the nursing  -Adherence to nursing standards  -Love of nursing  -Sense of purpose and worth being a nurse. | Having a strong professional identity | Traits related to the professional dimension |  |
| -Commitment to precise patient care  -Uphold human dignity in care  -Offer spiritual comfort to patients  -Deliver patient-centered care | Providing transcendent care |  |  |
| - Offer information for informed decisions  -Empower patients for health control  -Empowering patients to participate in their own care | paying more attention to patient education and empowerment |  |  |
| -Putting oneself in the patient's shoes  -Practice active listening and validation  -Understanding of patients' suffering | Deeper understanding of patients |  |  |
| - Mentor less experienced nurses/students  - Provide constructive feedback and encouragement to novice nurses/students  - Role model for professional behavior and value. | serving as mentors and role models for colleagues and nursing students |  |  |
| -Practice self-compassion and self-care  -Recognize and cope with distress  -Build resilience through reflection and support  -Develop healthy coping mechanisms | Increased resilience and adaptation | Traits related to personal dimension |  |
| -Address emotional needs with empathy  -Perceive and understand patient emotions  -Acknowledge and validate patients' feelings  -Express empathy through words and gestures | Increased empathy and compassion |  |  |
| - Enhance understanding of personal illness  - Increased self-management  -Incorporate personal illness experiences into nursing practice | post traumatic growth |  |  |
